# A cross-sectional examination of post-myocardial infarction physical activity levels among US rural and urban residents: Findings from the 2017–2019 Behavioral Risk Factor Surveillance System

**DOI:** 10.1371/journal.pone.0293343

**Published:** 2023-10-20

**Authors:** Phoebe Tran, Cristina Barroso, Liem Tran

**Affiliations:** 1 Department of Public Health, University of Tennessee, Knoxville, TN, United States of America; 2 College of Nursing, University of Tennessee, Knoxville, TN, United States of America; 3 Deparment of Geography and Sustainability, University of Tennessee, Knoxville, TN, United States of America; Northeastern University, UNITED STATES

## Abstract

**Background:**

This study sought to examine the relationship between rural residence and physical activity levels among US myocardial infarction (MI) survivors.

**Methods:**

We conducted a cross-sectional study using nationally representative Behavioral Risk Factor Surveillance System surveys from 2017 and 2019. We determined the survey-weighted percentage of rural and urban MI survivors meeting US physical activity guidelines. Logistic regression models were used to examine the relationship between rural/urban residence and meeting physical activity guidelines, accounting for sociodemographic factors.

**Results:**

Our study included 22,732 MI survivors (37.3% rural residents). The percentage of rural MI survivors meeting physical activity guidelines (37.4%, 95% CI: 35.1%-39.7%) was significantly less than their urban counterparts (45.6%, 95% CI: 44.0%-47.2%). Rural residence was associated with a 28.8% (95% CI: 20.0%-36.7%) lower odds of meeting physical activity guidelines, with this changing to a 19.3% (95% CI: 9.3%-28.3%) lower odds after adjustment for sociodemographic factors.

**Conclusions:**

A significant rural/urban disparity in physical activity levels exists among US MI survivors. Our findings support the need for further efforts to improve physical activity levels among rural MI survivors as part of successful secondary prevention in US high-MI burden rural areas.

## Introduction

Around 805,000 myocardial infarctions (MIs), also known as heart attacks, occur in the US annually, with disproportionately higher MI mortality rates found in rural (251.4 deaths/100,000 people) versus urban areas (208.6 deaths/100,000 people) [1–3]. Higher MI mortality rates in US rural areas may be attributed to significantly higher rates of cardiovascular disease risk factors such as physical inactivity, hypertension, diabetes, and obesity among rural versus nonrural populations [4]. Individuals with continued cardiovascular disease risk factors following the first MI face an increased risk of recurrent MI which is associated with 200–400% greater risk of mortality than the index MI [5, 6].

A jointly-issued American Heart Association and American College of Cardiology guideline for MI survivors recommends physical activity to aid recovery and prevent recurrent MI with the guideline specifying that clinicians treating MI survivors “should encourage 30 to 60 minutes of moderate-intensity aerobic activity, such as brisk walking, at least 5 days and preferably 7 days per week” [7]. Centers for Disease Control and Prevention (CDC) data from 2017 indicate that, among the general US adult population, a significantly lower percentage of rural (19.6%, 95% CI: 18.0%-23.1%) compared to urban residents (25.3%, 95% CI: 24.5%-26.2%) were physically active [8]. The limited research on rural/urban differences in physical activity levels following a cardiovascular disease (CVD) event has yielded conflicting results [9]. In addition, the existing literature focuses solely on stroke survivors whose health behaviors may differ from that of individuals affected by heart diseases such as MI [9, 10].

Accordingly, we used national US surveys to examine whether post-MI physical activity levels differed between rural and urban residents prior to and following adjustment for MI-related sociodemographic factors.

## Methods

### Study data and variables

For this study, we used cross-sectional Behavioral Risk Factor Surveillance System (BRFSS) surveys from years 2017 and 2019. These particular survey years were selected as the responses from the 2018 BRFSS physical activity variables cannot be combined with those from 2017 and 2019 and this avoids the influence of COVID-19 on exercise patterns during the 2020–2021 period. Conducted annually by the CDC, the BRFSS provides self-reported information on sociodemographic background, existing health conditions, and health-seeking behaviors of non-institutionalized US individuals aged ≥18 years [11]. The CDC administers the BRFSS to survey participants by landline or mobile phone [11].

Response rates for the BRFSS differ by state with rates ranging from 31%-64% in 2017 [12] and 37%-73% in 2019 [13]. With that being said response rates from the BRFSS are comparable if not better to those of other landline and cellphone-conducted surveys in the US such as the National Immunization Survey (landline response rate: 52%, cell phone response rate: 25%), Pew Research Center Internet Use Survey (landline response rate: 8.4%, cell phone response rate: 9.5%), and National Adult Tobacco Survey (landline response rate: 48%, cell phone response rate: 17%) [14]. BRFSS survey administrators rely on a combination of complex survey weights and oversampling of underrepresented groups (e.g. racial/ethnic minorities, rural residents) to ensure survey findings can be applied to the general US population [11]. BRFSS survey materials (i.e., codebooks, blank survey forms, survey methodology) can be accessed at the CDC’s website: https://www.cdc.gov/brfss/.

We identified MI survivors in the dataset through “Yes” responses to the BRFSS question, “(Ever told) you had a heart attack, also called a myocardial infarction?” [15]. Information on weekly physical activity levels was also obtained from the BRFSS. The BRFSS follows the US Department of Health and Human Services physical activity guidelines [16] to categorize survey respondents as inactive (no moderate-intensity physical activity/week), insufficiently active (<150 minutes of moderate-intensity physical activity/week), active (150–300 minutes of moderate-intensity physical activity/week), and highly active (≥300 minutes of moderate-intensity physical activity/week) [15]. For the purposes of our study, we considered active and highly active respondents to have met physical activity guidelines and insufficiently active and inactive respondents to have not met the guidelines. We used the BRFSS’s MSCODEs to determine rural/urban residence, classifying respondents with “not in a metropolitan statistical area (MSA)” responses as rural residents and those with “in the center city of an MSA”, “outside the center city of an MSA but inside the county containing the center city”, and “inside a suburban county of the MSA” responses as urban residents. Survey respondents were excluded if they were missing physical activity and MSCODE information. We also extracted data on MI- and physical activity-related sociodemographic factors (age, sex, race/ethnicity, income, education, health insurance, having a personal doctor) from the BRFSS; though unlike physical activity and MSCODEs, survey respondents were not excluded if they were missing covariate information.

### Ethics

BRFSS surveys have passed the CDC’s IRB and informed consent process. In addition, the CDC only provides deidentified surveys for public use. Moreover, the authors’ respective institution (The University of Tennessee Knoxville) categorizes the publicly available deidentified BRFSS data that was used for this study as falling under the public dataset exemption status, meaning further informed consent or IRB review is waived for researchers using this particular dataset.

### Statistical analyses

We conducted descriptive analyses to determine if the distribution of sociodemographic factors varied between rural and urban MI survivors. Following this, we used fractional hot-deck imputation, a method that allows us to fill in values for individuals with missing information on any of the abovementioned sociodemographic factors (n = 4457, 19.6% of dataset) while also accounting for survey weighting [17]. In hot deck imputation, the individual with a missing value on say variable X (recipient) has it replaced with the observed variable X value of an individual (donor) who has similar values to the recipient on observed variables Y and Z [18]. Fractional hot deck imputation is a type of hot deck imputation where instead of one donor’s observed value replacing the missing value for a recipient, multiple donors with similar characteristics to the recipient will contribute to replacing the recipient’s missing value by a fraction of the original weight of the recipient [18]. The prevalence of post-MI physical activity levels among rural and urban residents was then assessed.

We used logistic regression models to examine the relationship between rural residence and physical activity prior to and following adjustment for sociodemographic factors (age, sex, race, income, education, health insurance, and having a personal doctor). We selected these particular sociodemographic factors to adjust for in the logistic regression model based on (1) prior evidence from the cardiovascular disease (CVD) and exercise literature that these covariates are potential confounders that are associated with CVD and rural/urban status and (2) our own bivariate analyses that these covariates (age, sex, race, income, education, health insurance, and having a personal doctor) are significantly associated with physical activity at α = 0.10 [3, 19–21]. As part of supplemental analysis, we also considered whether (1) age, sex, and race/ethnicity moderated the adjusted association between rural residence and physical activity levels and (2) if the intensity and duration of physical activity (assessed using the inactive, insufficiently active, active, and highly active categories) differed between MI survivors in rural and urban areas (**S1–S4 Tables**). All analyses were conducted in SAS Version 9.4, using the SAS survey procedures to account for the BRFSS’s survey weighting. All statistical testing was 2-sided and conducted at α = 0.05.

## Results

The study included 22,732 BRFSS survey respondents who had MI (**Table 1**). Of these MI survivors, 37.3% were rural residents. Rural MI survivors were more likely to be White, have a lower income, and completed fewer years of education compared to those in urban areas.

**Table 1:** Characteristics of myocardial infarction survivors in the 2017 and 2019 Behavioral Risk Factor Surveillance System (BRFSS) surveys by rural residence status (N = 22,732).

Covariates	Rural (n = 8478)		Urban (n = 14254)	
	Frequency (n)	Weighted %^1^	Frequency (n)	Weighted %
		(95% Confidence Interval)		(95% Confidence Interval)
Physical activity				
Met physical activity recommendations	3531	37.4 (35.1, 39.7)	6517	45.6 (44.0, 47.2)
Did not meet physical activity recommendations	4947	62.6 (60.3, 64.9)	7737	54.4 (52.8, 56.0)
Age				
18–44 years	106	2.6 (1.8, 3.4)	161	2.6 (2.0, 3.3)
45–64 years	1886	29.4 (27.2, 31.7)	2688	24.6 (23.1, 26.0)
65 years and up	6486	68.0 (65.7, 70.2)	11405	72.8 (71.3, 74.3)
Sex				
Male	4576	55.9 (53.5, 58.3)	7669	56.4 (54.8, 58.0)
Female	3902	44.1 (41.7, 46.5)	6585	43.6 (42, 45.2)
Race				
White	7281	86.2 (84.8, 87.7)	11624	75.2 (73.6, 76.7)
Black	320	5.4 (4.4, 6.4)	1225	10.5 (9.4, 11.6)
Other	715	6.3 (5.3, 7.2)	1108	12.0 (10.7, 13.3)
Missing	162	2.1 (1.5, 2.6)	297	2.4 (1.9, 2.9)
Income				
Less than $15,000	1244	15.3 (13.6, 17.1)	1500	10.4 (9.3, 11.4)
$15,000 to less than $25,000	1874	22.4 (20.4, 24.5)	2690	19.3 (18, 20.6)
$25,000 to less than $35,000	1003	13.2 (11.4, 14.9)	1617	11.2 (10.2, 12.3)
$35,000 to less than $50,000	1097	11.6 (10.4, 12.9)	1908	12.5 (11.5, 13.4)
$50,000 or more	1744	17.4 (15.9, 19.0)	3974	27.9 (26.4, 29.3)
Missing	1516	20.0 (17.9, 22)	2565	18.7 (17.5, 20.0)
Education				
Did not complete high school	1105	24.5 (22.1, 26.8)	1358	17.8 (16.3, 19.3)
High school graduate	3165	38.1 (35.9, 40.3)	4456	31.3 (29.8, 32.7)
Some college or technical school	2368	26.4 (24.3, 28.5)	4154	31.8 (30.3, 33.3)
College graduate	1840	11.0 (10.1, 12.0)	4286	19.1 (18.1, 20.2)
Health insurance				
Yes	8168	95.7 (94.6, 96.9)	13862	95.5 (94.7, 96.3)
No	281	3.9 (2.7, 5.0)	359	4.3 (3.5, 5.1)
Missing	29	0.4 (0.1, 0.8)	33	0.2 (0.1, 0.3)
Personal doctor				
Yes, only one	6725	79.2 (77.3, 81.1)	11522	79.4 (78.2, 80.7)
More than one	1179	14.4 (12.8, 16.0)	2026	14.8 (13.7, 15.9)
No	539	5.6 (4.6, 6.6)	649	5.3 (4.6, 6.1)
Missing	35	0.8 (0.2, 1.4)	57	0.4 (0.2, 0.6)

^1^ BRFSS survey-weights have been used to calculate all weighted percentages in the table.

**Fig 1:**
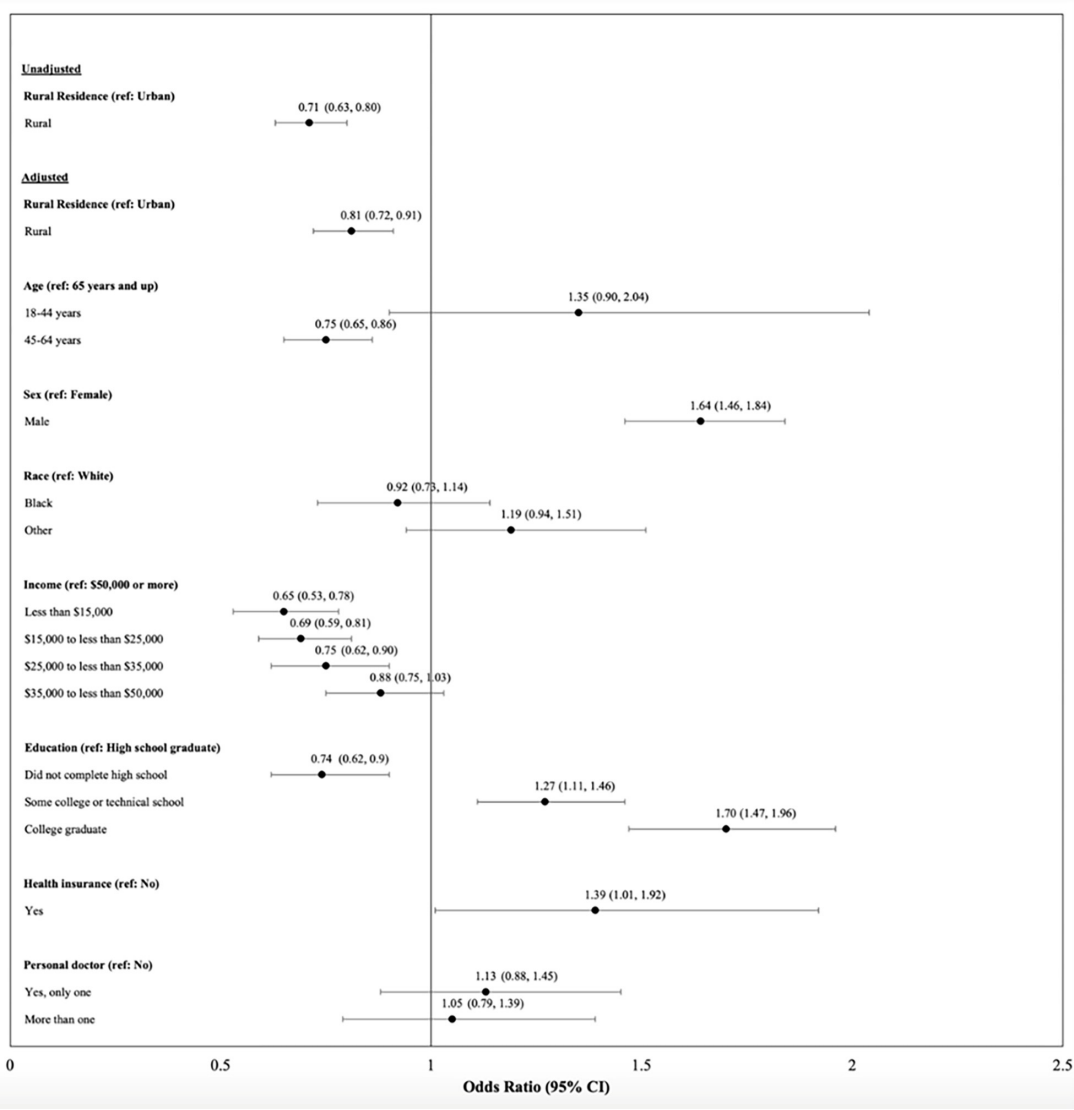
Associations between rural residence and meeting physical activity guidelines among US myocardial infarction survivors.

The percentage of rural MI survivors meeting physical activity guidelines (37.4%, 95% CI: 35.1%-39.7%) was significantly less than that of urban MI survivors (45.6%, 95% CI: 44.0%-47.2%) **(Table 1)**. Among MI survivors, rural residence was significantly associated with a 28.8% (95% CI: 20.0%-36.7%) lower odds of meeting physical activity guidelines **(Fig 1)**. Although this association became slightly attenuated following adjustment for sociodemographic factors, rural residence was still significantly associated with a 19.3% (95% CI: 9.3%-28.3%) lower odds of meeting physical activity guidelines.

Other significant predictors of meeting physical activity guidelines included age, sex, income, education, and insurance. Independent of rural residence, being male, having higher income, and completing more years of education corresponded with greater odds of meeting physical activity guidelines. The relationship between age and meeting physical activity guidelines was more mixed with individuals aged 18–44 years being more likely to meet guidelines than those 65 years and up and those 45–64 years less likely to meet guidelines than those 65 years and up. Our supplemental analyses indicated that neither the age and physical activity, sex and physical activity, nor race/ethnicity and physical activity interaction terms were significant. However, supplemental analyses did show that intensity and duration of physical activity was significantly less among rural versus urban MI survivors, results that are in line with our findings on meeting physical activity guidelines.

## Discussion

We conducted a nationally representative study examining differences in contemporary post-MI physical activity levels between US rural and urban residents. Less than 40% of rural MI survivors met physical activity guidelines. In addition, rural residence was significantly linked with lower odds of MI survivors meeting physical activity guidelines prior to and following adjustment.

Prior research examining rural/urban differences in physical activity levels after a CVD event has been limited [9, 10]. In a study of Canadian individuals enrolled in the Cardiovascular Health in Ambulatory Care Research Team (CANHEART) cohort between 2008–2012, investigators found that stroke survivors in rural areas were more likely to be physically active (48%) than those in urban areas (33%) [10]. In contrast, the sole US study of rural/urban differences in post-CVD physical activity levels reported that, among 2019 BRFSS survey respondents, rural stroke survivors were less likely to be physically active (29.8%, 95% CI: 25.4%-34.2%) than their urban counterparts (32.5%, 95% CI: 29.8%- 35.2%) [9]. Regarding the sociodemographic factors that were considered, our findings generally align with those from a integrative review of physical activity among CVD patients that reported male sex and higher income to be significant predictors of higher physical activity levels [22]. Our findings expand on previous work by providing physical activity level estimates specific to US rural and urban MI survivors, and further evidence of a US rural/urban disparity in physical activity levels following a CVD event.

Although we found slightly higher post-MI physical activity levels than what has been previously documented for stroke, the percentage of US MI survivors meeting physical activity guidelines remains particularly low with a rural-urban divide in post-MI physical activity levels evident. Rural areas lack public features such as parks and sidewalks that would make it easier for residents to engage in physical activity. One study found that the median distance to a park in US rural and urban areas was 6.2 miles and 0.5 miles respectively [23]. According to the US Health Resources and Services Administration, the lack of parks and sidewalks in US rural areas may be partly attributed to these regions having a smaller tax base to fund this public feature [24]. Conversely, the influence of rural areas’ limited parks and sidewalks availability on post-MI physical activity may be mitigated by the fact US rural areas have a higher median percentage of green space (54.9%) than US urban areas (2.8%) [23].

Furthermore, the few available rural specific physical activity interventions may be a contributor to the rural-urban physical activity divide. With regard to rural-specific physical activity interventions conducted in the US, a 2019 review identified only 29 coming from distinct studies [25]. Identified interventions included incentivized physical activity programs, mobile health delivered exercise reminders, goal setting, and self-monitoring [25]. None of the identified interventions were tailored to CVD survivors [25]. With that being said, increased availability of rural public features (i.e., parks, sidewalks) conducive to physical activity through governmental investment and development of interventions specific to CVD survivors may assist more rural MI survivors to incorporate physical activity into their secondary prevention strategies.

We consider study limitations below. As the physical activity outcome is self-reported, there is the potential for self-report and non-response bias to be present. While these types of biases cannot be fully excluded from self-reported data, there has been extensive research from the CDC and non-CDC affiliated investigators documenting good correlation between BRFSS responses and measurements collected in-person [26]. Due to BRFSS being cross-sectional, questions related to timing of health conditions or health behaviors cannot be addressed. Despite the cross-sectional nature of the BRFSS, this dataset allows for examination of nationally representative patterns of post-MI physical activity with questions on timing of post-MI physical activity initiation to be explored in future longitudinal studies. Our study does not include MI survivors aged <18 years or those that are institutionalized. However, as MIs in children are rare and the majority of US individuals are not institutionalized, our findings should be applicable to most US MI survivors.

## Conclusion

This study showed that a large proportion of US rural MI survivors are not meeting physical activity guidelines even after accounting for sociodemographic factors, an area of concern relating to successful secondary MI prevention. This research expands understanding of US rural/urban differences in post-CVD physical activity levels, providing knowledge supporting the need for further physical activity-related secondary prevention efforts in high-MI burden rural areas across the country.

## Supporting information

S1 Table Associations between rural residence and meeting physical activity guidelines among US myocardial infarction survivors with rural residence and age interaction term. (DOCX)

S2 Table Associations between rural residence and meeting physical activity guidelines among US myocardial infarction survivors with rural residence and sex interaction term. (DOCX)

S3 Table Associations between rural residence and meeting physical activity guidelines among US myocardial infarction survivors with rural residence and race/ethnicity interaction term. (DOCX)

S4 Table Distribution of physical activity categories among US myocardial infarction survivors in rural and urban areas. (DOCX)
