# Supplementary material for: A cross-sectional examination of post-myocardial infarction physical activity levels among US rural and urban residents: Findings from the 2017–2019 Behavioral Risk Factor Surveillance System
Source: PLoS One. 2023 Oct 20;18(10):e0293343. doi: 10.1371/journal.pone.0293343 (PMC10588872; doi:10.1371/journal.pone.0293343)
Supplement: S2 Table — (DOCX) [file pone.0293343.s002.docx]

**S2 Table. Associations between rural residence and meeting physical activity guidelines among US myocardial infarction survivors with rural residence and sex interaction term.**

| Covariates | Adjusted | |
| --- | --- | --- |
|  | **OR (95% CI)** | **p value** |
| Rural Residence |  | 0.0013 |
| Rural | 0.78 (0.67, 0.91) |  |
| Urban | Ref |  |
| Age |  | <0.0001 |
| 18-44 years | 1.35 (0.90, 2.04) |  |
| 45-64 years | 0.75 (0.65, 0.86) |  |
| 65 years and up | Ref |  |
| Sex |  | <0.0001 |
| Male | 0.60 (0.52, 0.69) |  |
| Female | Ref |  |
| Race |  | 0.2401 |
| White | Ref |  |
| Black | 0.92 (0.73, 1.15) |  |
| Other | 1.19 (0.94, 1.51) |  |
| Income |  | <0.0001 |
| Less than $15,000 | 0.65 (0.53, 0.78) |  |
| $15,000 to less than $25,000 | 0.69 (0.59, 0.81) |  |
| $25,000 to less than $35,000 | 0.75 (0.62, 0.90) |  |
| $35,000 to less than $50,000 | 0.88 (0.75, 1.03) |  |
| $50,000 or more | Ref |  |
| Education |  | <0.0001 |
| Did not complete high school | 0.74 (0.62, 0.90) |  |
| High school graduate | Ref |  |
| Some college or technical school | 1.27 (1.11, 1.46) |  |
| College graduate | 1.69 (1.47, 1.96) |  |
| Health insurance |  | 0.0425 |
| Yes | 1.40 (1.01, 1.93) |  |
| No | Ref |  |
| Personal doctor |  | 0.4605 |
| Yes, only one | 1.13 (0.88, 1.44) |  |
| More than one | 1.05 (0.79, 1.39) |  |
| No | Ref |  |
| Rural Residence*Sex |  | 0.5246 |
| Rural*Male | 1.08 (0.86, 1.36) |  |
