# Supplementary material for: A cross-sectional examination of post-myocardial infarction physical activity levels among US rural and urban residents: Findings from the 2017–2019 Behavioral Risk Factor Surveillance System
Source: PLoS One. 2023 Oct 20;18(10):e0293343. doi: 10.1371/journal.pone.0293343 (PMC10588872; doi:10.1371/journal.pone.0293343)
Supplement: S4 Table — (DOCX) [file pone.0293343.s004.docx]

**S4 Table. Distribution of physical activity categories among US myocardial infarction survivors in rural and urban areas**

|  | **Rural** | **Urban** | **Chi Square comparing rural versus urban for different physical activity levels** |
| --- | --- | --- | --- |
|  | **Weighted %** | **Weighted %** | **P value** |
| **Highly Active** | 8.5 | 13.0 | <.0001 |
| **Active** | 28.9 | 32.7 | <.0001 |
| **Insufficiently Active** | 51.9 | 43.2 | 0.0159 |
| **Inactive** | 10.7 | 11.1 | Reference |

^1^Overall Chi square test p value was <.0001.
